# *rpavirtual*: Key lessons in healthcare organisational resilience in the time of COVID‐19

**DOI:** 10.1002/hpm.3430

**Published:** 2022-02-10

**Authors:** Miranda Shaw, Teresa Anderson, Tim Sinclair, Owen Hutchings, Cassandra Dearing, Freya Raffan, Dianna Jagers, David Greenfield

**Affiliations:** ^1^ RPA Virtual Hospital Sydney Local Health District Camperdown New South Wales Australia; ^2^ Sydney Local Health District Camperdown New South Wales Australia; ^3^ Australian Institute of Health Service Management University of Tasmania Sydney New South Wales Australia

**Keywords:** COVID‐19, healthcare organisational resilience, hospital, safety and quality, virtual care

## Abstract

The COVID‐19 pandemic is an unfolding crisis which is continually testing the resilience of healthcare organisations. In this context, a key requirement for executives, managers and frontline staff is continually adapting, learning and coping with complexity under pressure to deliver high quality and safe care. Sydney Local Health District has responded to the COVID‐19 crisis, in part, through the pivoting of *rpavirtual*, a newly established virtual health service, to deliver an innovative model of care in a clinically rigorous and safe manner. Through reviewing the rapid evolution of *rpavirtual'*s purpose, implementation challenges and impact, we investigate how it has displayed resilience and derive key lessons for health organisations.

## Highlights

- The COVID‐19 pandemic continues to test healthcare organisational resilience.
- Resilience is interconnected and reinforced at all levels of health care.
- Resilience is adapting, learning and coping with complexities under pressure.
- High quality and safe care can be delivered through virtual technologies.

## 1 BACKGROUND

The COVID‐19 pandemic has tested healthcare organisations like few other crises in living memory. In this extraordinary context, resilient and reliable healthcare organisations are marked by their capacity for learning and continual improvement.^1^, ^2^ These organisations display seven properties: top‐level commitment; just culture; learning culture; awareness; flexibility; preparedness and opacity.^1^, ^2^, ^3^ They embody the ability to be able to rapidly change, with a minimum of disruption, adapting to external and internal influences. They have preventative and adaptive capacities to learn, adapt and cope with complexity under pressure to achieve safety.^1^, ^2^, ^4^ Resilience is interconnected, via a systems‐based approach, at three levels: individual, interprofessional and organisational.^1^, ^2^, ^5^

Sydney Local Health District (SLHD) began to establish in February 2020 a new hospital service, a ‘virtual hospital’—*rpavirtual—*in the densely built‐up, populated metropolitan inner city of Sydney, Australia. The new virtual service was intended to use a sustainable, scalable model of integrated care that harnessed ehealth technologies, to meet consumer expectations for more flexible care delivery. SLHD established *rpavirtual* to complement existing hospital and community‐based services, including a primary referral hospital at Royal Prince Alfred (RPA). The SLHD is widely recognised as a leader in research, education and in developing innovative models of care.^6^

Within 4 weeks of launch, *rpavirtual* was required to rapidly scale‐up to support the SLHD response to the COVID‐19 pandemic. *rpavirtual* was tasked to provide care for all COVID‐19 positive patients in either home isolation or a ‘health hotel’ within the district boundaries. This necessitated *rpavirtual* redirecting its focus and rapidly developing new virtual clinical care models. This significant change was within the context of an unpredictable, emerging COVID‐19 disease spread and clinical uncertainty. Additionally, in this highly emotionally charged climate the health workforce was reassigned to *rpavirtual* into new roles, different from their usual employment.

In this context *rpavirtual* adapted, learnt and coped with complexity under pressure to deliver high quality and safe care. Through reviewing the rapid evolution of *rpavirtual*'s purpose, implementation challenges and impact, we investigate how it has displayed resilience and derive key lessons for health organisations.

## 2 *rpavirtual*: INITIAL FOCUS

*rpavirtual* was established on the basis that virtual models of care increase patient and carer satisfaction by supporting patients to remain in their home, rather than in a hospital, or to remain in a local hospital with specialised support provided from a distance.^7^ In‐home nursing care delivered in ‘virtual’ patient beds supports avoidable hospital presentations including readmissions, early discharge from hospital and complements primary care.^7^

SLHD had no blueprint to work from when developing *rpavirtual*. Hence, the initial emphasis was on developing necessary infrastructure and extensive consultation to inform the initial model of care. The infrastructure established was an integrated soci‐technical system suitable for a virtual care facility.^8^ This included an executive team and clinical leadership with integrated corporate and clinical governance systems; a virtual nursing care centre; a flexible, scalable location and care model; and ehealth, medical record, videoconferencing and telephone systems. Collaboration work focussed on identifying opportunities with clinical specialties and primary care. General practice was at the centre of this endeavour providing overarching medical governance for patients which supported a continuum of care model.

The governance structure for *rpavirtual* was integrated into the SLHD. The *rpavirtual* leadership team, comprising a General Manager, Clinical Director and Director of Nursing, has significant shared experience in community health, general practice and hospital settings. This team is supported by a Steering Committee, Evaluation Committee, Research Committee and a Clinical Advisory Council. Membership of the Clinical Advisory Council includes SLHD Senior Executive Directors as well as medical, nursing and allied health clinical and informatics professionals. The New South Wales Ministry of Health and the Australian Commission on Safety and Quality in Health Care are represented on the steering and evaluation committees.

Controlled expansion of *rpavirtual*'s patient cohort was planned to build on the 100 patients initially enrolled, which included patients receiving home palliative care, adults with cystic fibrosis and other patient groups with chronic conditions with a history or substantial risk of frequent emergency department presentation. *rpavirtual* incorporated into their virtual hospital programme the established community nursing service, known as Sydney District Nursing. This service supports over 1000 patients in their homes at any one time, including those requiring: chronic and complex care, including complex wound care; palliative care and end‐of‐life care; and hospital‐in‐the‐home. From this modest beginning, a progressive roll‐out of the programme was intended. The aim, to be achieved over a 12‐month proof‐of‐concept trial period, was to grow and develop a 2000 virtual bed hospital and integrate it with the established 1000+ patient community nursing service.

## 3 *rpavirtual* PIVOTING TO RESPOND TO COVID‐19

**FIGURE 1:**
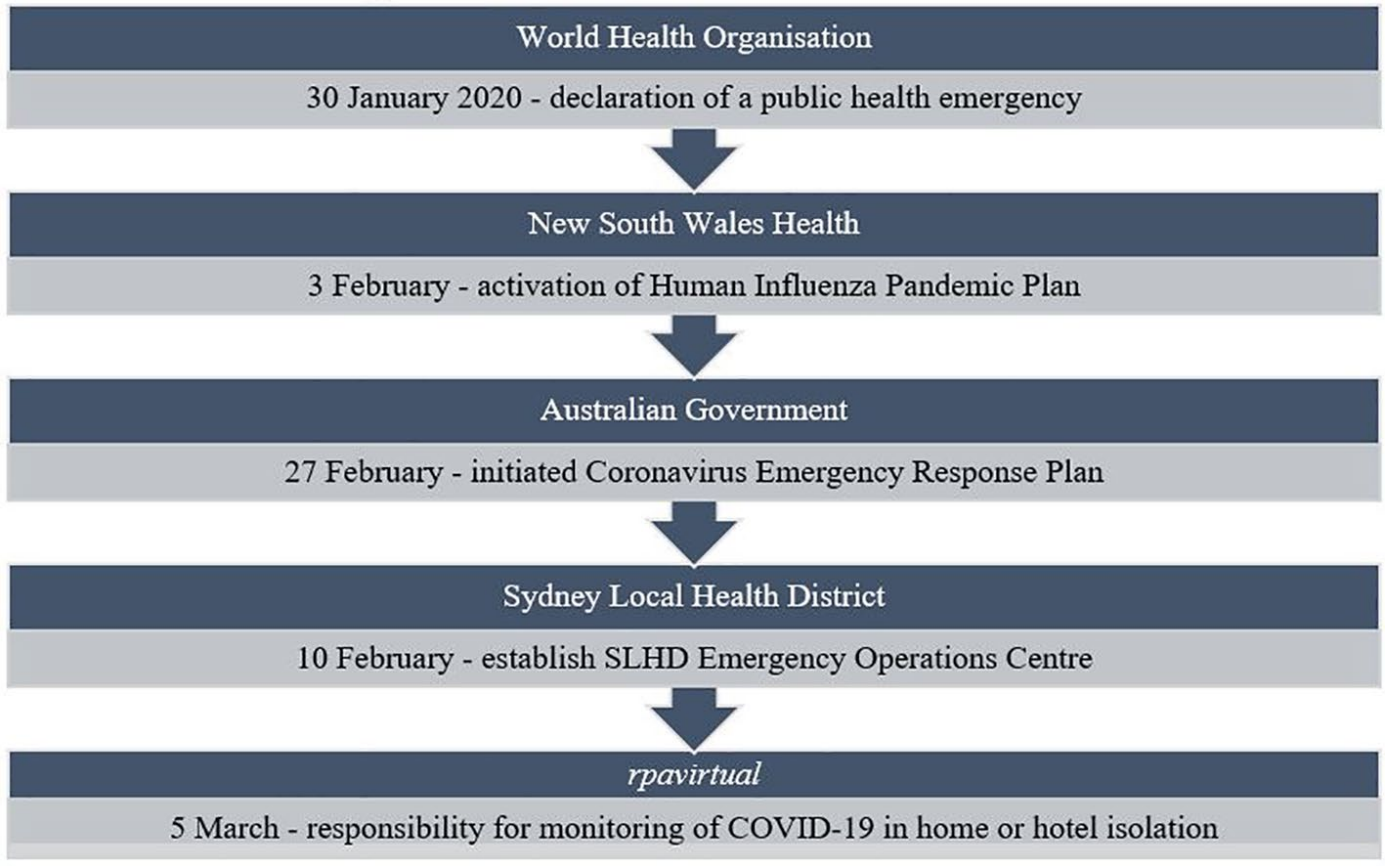
COVID‐19 organisational response timeline

In early 2020 in the space of 5 weeks, COVID‐19 was identified as a global pandemic by the World Health Organisation, which initiated state and national health response plan activation and, in turn, action by the SLHD (Figure 1). *rpavirtual* was tasked to pivot from its existing focus to urgently develop a model for virtual monitoring of stable adult patients with COVID‐19 in home isolation. At the time of initial implementation planning, the scale of the COVID‐19 pandemic in Australia was unknown. At the start of March, there were 22 COVID‐19 patients in NSW. On the 11 March, when the WHO declared COVID‐19 a pandemic, there were 77 COVID‐19 patients in NSW. The Australian Commonwealth Department of Health, on 13 March 2020, introduced national funding to support flexible virtual and telehealth services.^9^

*rpavirtual* commenced virtual care of COVID‐19 patients in home isolation on 11 March. Whilst *rpavirtual* was initially tasked with clinical governance of all COVID‐19 positive patients in home isolation and in SLHD health hotels, the service remit was broadened to also provide clinical care for returning travellers in enforced quarantine who are COVID‐19 negative. These patients are presenting with more complex health issues which has necessitated the introduction of further virtual models of care such as antenatal care, paediatrics, mental health, drug and alcohol, and aged care.

To date, there have been 3479 cases of COVID‐19 diagnosed in New South Wales.^10^ By mid‐July 2020, 691 patients were registered with *rpavirtual*, of which 40 required escalation for hospitalisation following clinical deterioration and 18 were admitted to a critical care ward. However, the majority of the cohort, 596 patients, have recovered and been discharged from the service.

*rpavirtual* with soci‐technical infrastructure systems in place, was able to immediately pivot the focus of care to COVID‐19 patients. The *rpavirtual* executive team met daily to discuss the evolving understanding of COVID‐19 and the implications for clinical workforce needs and capacity, clinical care issues, resourcing, equipment logistics and technology requirements. To develop and continually refine a virtual monitoring model of care immediate and ongoing consultation occurred with medical teams and specialists in respiratory, infectious diseases, emergency medicine and public health. A key need identified was the requirement for additional medical and nursing staff to assist with implementation of virtual monitoring of COVID‐19 patients. This necessitated the relocation of 20 acute hospital registered nurses and 3 medical registrars to the *rpavirtual* Care Centre, many of whom were considered vulnerable to COVID‐19 infection.^11^

## 4 DEVELOPING AND IMPLEMENTING A *rpavirtual* COVID‐19 MODEL OF CARE

*rpavirtual* designed, implemented and has been continually revising a unique COVID‐19 model of care and clinical monitoring protocol (Table 1). The ongoing refinement is occurring as knowledge about the virus develops and experience with the model of care increases. The model of care enacted a rigorous, standardised patient selection criteria, effective monitoring and identification of clinical deterioration, including hypoxia irrespective of symptoms, and discharge process. *rpavirtual* COVID‐19 model of care inclusion, exclusion and discharge criteria and process were established to ensure only those patients sufficiently stable and with minimal risk factors were managed by the service.

**TABLE 1:** *rpavirtual* model of care

*rpavirtual* model of care	
Inclusion criteria	Exclusion criteria
Return a positive result for COVID‐19	Over 65 years with comorbidity including, cancer, cardiovascular disease, diabetes, heart failure, immunosuppression, stroke, liver disease, renal disease and lung disease
Clinical team assessment that patient can be managed through remote monitoring	Under 65 years with one or more of the following comorbidities: lung disease, cardiovascular disease, renal disease (level 5 renal failure)
Patient can self‐isolate safely and understands how to manage self‐isolation	Residents of residential aged care facilities
Care can be by patient self‐care or by familial carer using PPE	
Case‐by‐case assessment of individual patients, in consultation with specialists:	
With managed hypertension	
Pregnant women	
Children	

COVID‐19 positive patient risk stratification		
Low risk	Moderate risk	High risk
Under 50 years	Under or over 50 years	Relevant medical history
Asymptomatic or mild symptoms	Moderate symptoms:	<65 years – lung disease, cardiovascular disease or renal disease (level 5 renal failure)
No symptoms:	Fever >38	>65 years – cancer, cardiovascular disease, hypertension, diabetes, heart failure, immunosuppression, stroke, liver disease, renal disease or lung disease
Low grade fever <38	Marked cough/sputum	
Mild cough and upper respiratory tract symptoms	Shortness of breath/may find it difficult to talk	
No or minimal breathlessness		

Clinical monitoring process	
Patient support, self‐monitoring and access to services	Clinical team monitoring
Ongoing 24/7 telephone/video access to *rpavirtual* Care Centre	Vital signs monitoring through patient wearables and supplemented with clinical assessment using video; temperature is continuously monitored and relayed to an app with built in alert parameters
Self‐monitoring of physical and emotional/psychological states	Vital signs of temperature, pulse and oxygen saturation rate are recorded three times daily in electronic medical record
Access to initiate ambulance retrieval to hospital	Clinical assessment conducted twice‐daily using videoconferencing; assessing patient symptoms, question and observe respiratory rate, level of anxiety, physical appearance and overall wellbeing

Low risk	Medium risk	High risk
Day 1—patient receives initial video consult from *rpavirtual* and information on how to escalate if they start to deteriorate	Day 1—patient receives initial video consult from *rpavirtual* and information on how to escalate if they start to deteriorate between contacts	Day 1— patient receives initial video consult from *rpavirtual* and information on how to escalate if they start to deteriorate between contacts
Day 3—patient is contacted via videoconference for clinical assessment	Day 3—patient is contacted via videoconference for clinical assessment	Wearable devices are delivered to patient home
If at day 3 patient shows signs of deterioration not requiring ED, re‐categorise as **medium risk** and provide DAILY videoconference; consider need for remote monitoring	Day 6–10—patient is contacted daily via videoconference for clinical assessment	Three times per day – video call from *rpavirtual* which includes patient reported observations and clinical assessment
Day 10—patient receives video call from *rpavirtual* for consideration of discharge	After day 10, patient is contacted every 3 days until ready for discharge	Patient is contacted three times per day until ready for discharge
Patient is contacted every 3 days until ready for discharge	If at any time patient is becoming more unwell, re‐categorise as **high risk**	Consider need for increased support based on carer/nurse availability
Patient discharge criteria		
At least 7 days after the onset of the acute illness		
Resolution of the acute illness for the previous 24 h		
Afebrile for the previous 48 h		
PCR negative results on at least two consecutive respiratory specimens collected 24 h apart after the acute illness has resolved for patients are a health worker or a worker in aged care		
Staff wellbeing and safety issues		
Implementation of established organisational infection control practices, known and practiced by staff		
Online and practical training, initial course and refresher updates, in use of PPE and patient/family interactions		
Staff screening upon arrival at work each day including temperature check		
Standardised home visiting risk assessment including questions related to COVID‐19 transmission risk and a requirement for COVID patients and household members to wear PPE		
Monthly staff information webinars, regular Chief Executive updates and weekly NSW Health COVID‐19 newsletter updates		
Availability and promotion of an internal Employee Assistance (psychology) Service		
*rpavirtual* Care Centre roles available to staff considered vulnerable to COVID‐19 infection		
Staff celebrations for key milestones		
Distribution of positive results and patient feedback		
Complaints handling education and clear escalation pathways		

Wearable ehealth devices for the measurement of vital signs, specifically a temperature probe and pulse oximeter, were adopted. Selection of specific items was guided by the key considerations of clinical relevance, ease of use, compatibility with organisational information technology systems, availability, and cost. All monitoring devices are single use only and are delivered to patient homes free of charge.

The clinical review process recognised the potential for undetected deterioration by external clinical assessment; hence the protocol incorporated patient ongoing self‐assessment, referral and escalation. The assessment process adopted the SLHD ‘Between the Flags’ programme^12^ and patient management and escalation procedures, well established and known by staff, to promote appropriate, timely and safe responses. In the event of deterioration, patients are directed to RPA Hospital Emergency Department. If clinically indicated transfer is via ambulance, who are notified of the patient's infectious status and advised to call the Emergency Department prior to arrival.

The first 100 COVID‐19 patients were provided with wearable devices and twice daily clinical assessment. However, to prepare for the potential ongoing demand and/or escalation in the volume of patients, and ensure sustainable allocation of limited resources, a patient risk assessment step was subsequently introduced. Patients were risk stratified as low, medium or high risk for deterioration and a level of care prescribed accordingly. Now, only high‐risk patients are provided with wearable devices and thrice‐daily clinical assessment. Low and moderate risk patients participate in virtual assessments at less frequent intervals, according to a detailed clinical protocol. As a safety net all patients have 24/7 access to the *rpavirtual* Care Centre in the event that their condition deteriorates and/or with any concerns.

Discharge criteria was directed by a national health policy guideline ‐ the Australian Health Protection Principal Committee COVID‐19 Statement (released 21 March 2020 and updated).^13^ A COVID‐19 discharge form was introduced into the electronic medical record. This form is completed by the treating medical officer and reviewed and endorsed by the *rpavirtual* Clinical Director. At discharge the form is electronically mailed, using secure file transfer, to the patient and their general practitioner.

To undertake clinical care work health professionals were also required to visit patients/families at home. The visits were to provide supplies of personal protective equipment (PPE), kits of wearable devices for clinical monitoring, and conduct health and emotional assessments. Hence, to ensure the physical health and emotional wellbeing of staff *rpavirtual* instigated a range of measures. The aim was to maximise workforce protection from patient or environmental infection transmission risks.^10^ As important, this programme also attended to the staff emotional and psychological anxiety and stress associated with COVID‐19.

Patient experience data was collected and used as part of the process of assessing and refining *rpavirtual* service delivery. Patient experience surveys were developed, based on existing surveys endorsed by NSW Health,^14^ and modified slightly to reflect the virtual care environment. Topics addressed included experience of service and care; communication with health professionals; and impact of use of technology. Surveys were distributed via SMS messaging, and patients completed the questionnaire in a secure web application. The initial patient cohort returned 215 completed surveys, a response rate of 54% for the period 23 March–30 June 2020. Patients reported positively, and at the same time that improvements could be made to, the service processes, communication from the health professionals and the use of technology. A typical response from patients is reflected in the following statement: ‘The technology and the excellent interpersonal skills of the staff. It is an awesome arrangement and all involved should be congratulated. It provides great reassurance to patients at a time of stress’.

## 5 LESSONS LEARNT

A key lesson from SLHD *rpavirtual* response to COVID‐19 is that healthcare resilience is interconnected and reinforced at organisational, interprofessional and individual levels.^5^ SLHD faced the critical necessity to respond, in a flexible, timely manner to COVID‐19 and provide safe, high‐quality services; the pandemic was the ‘burning platform’ required to affect immediate change within the organisation driving the top‐level commitment to do so. *rpavirtual* care shifted from being a concept or idea for many services, teams and clinicians to the actual modality of care required in this unpredictable, unfolding context. The external environmental needs rapidly accelerated the internal cultural change processes, stimulating innovation, learning and continual improvement activities. The SLHD's well‐established and integrated structure and systems for governance—both organisational and clinical—enabled the rapid change; simultaneously, the governance arrangements embodied a just and learning culture effective for achieving positive outcomes. Hence, the second key lesson is that the pandemic crisis is continually testing the SLHD organisational resilience and highlighting underlying strengths and areas for improvement in governance operations.

The third key lesson is that healthcare resilience is established and reinforced through interprofessional collaboration and team working. *rpavirtual* required professionals with clinical, administrative and technology expertise to together design, deliver and continually improve integrated virtual care models. Professionals, both from within SLHD and those in associated primary care services, with situational awareness, flexibility and preparedness enabled this outcome.^2^, ^3^ Collectively and individually, professionals with these characteristics adapted and facilitated *rpavirtual* into reality. In a dynamic, unpredictable context an organisational governance system that promotes and sustains engagement, communication, accountability and transparency is critical. Supported by efficient governance systems, reinforced by staff physical health and wellbeing strategies, disparate teams of redeployed staff within days developed into collaborative, effective teams. In doing so, the *rpavirtual* model of care has been shown to be an essential component of ongoing health infrastructure—it offers a highly adaptable, scalable, translational, and technologically and clinically effective service.

The fourth key lesson is at the individual level for the clinician–patients dyad. High‐quality services, using continually monitored and refined models of care which extend the established organisational soci‐technical infrastructure, can be delivered effectively to meet both clinician and patient expectations. In short, clinician assessment, monitoring and treatment within the hospital context can be delivered safely via technology in the community environment; and, patients accept and respond well to comprehensive, supportive care delivered through virtual technologies.

## 6 CONCLUSION

The COVID‐19 pandemic is an unfolding crisis which is continually testing the resilience of healthcare organisations. The key lessons derived from the introduction of *rpavirtual* demonstrate that healthcare resilience is interconnected and reinforced at organisational, interprofessional and individual levels. Organisations, such as *rpavirtual*, that achieve and maintain cohesion across these levels learn, adapt and provide safe, high‐quality care. The *rpavirtual* model was conceived and developed as a purpose designed service embedded within a Local Health District. A comprehensive and unique governance structure operates in accordance with the general and clinical governance expectations of the health organisation. *rpavirtual* acts as both an extension to existing services as well as providing new virtual models of care for specific patient cohorts and conditions. The medical support provided to patients is supplemented where necessary by drawing on the existing specialist services. Leadership, good management and comprehensive clinical governance were critical to establishing the infrastructure required for *rpavirtual* to deliver new virtual models of care that were safe and met the expectations of patients and clinicians.

## CONFLICT OF INTEREST

The authors have no conflict of interest to declare.

## ETHICS STATEMENT

No ethical approval from the institutional ethics review committee was needed.

## Data Availability

The data that support the findings of this study are available from the corresponding author upon reasonable request.

## References

[1] Babyar J . Direct reliability: strategies to revolutionize healthcare. J Publ Health. 2020;28(1):89‐95.

[2] Sujan MA . High reliability organisations: making care safer through reliability and resilience. In: Baillie L , Maxwell E , eds. Improving Healthcare – A Handbook for Practitioners. Routledge; 2017.

[3] Wreathall J . Properties of resilience organisations: an initial view. In: Hollnagel E , Woods D , Leveson N , eds. Resilience Engineering: Concepts and Precepts. Ashgate; 2006:275‐285.

[4] Hollnagel E , Woods D , Leveson N . Resilience Engineering: Concepts and Precepts. Ashgate; 2006.

[5] Jeffcott S , Elbraahim E , Cameron P . Resilience in healthcare and clinical handover. BMJ Qual Saf. 2008;18(4):256‐260. 10.1136/qshc.2008.030163 19651927

[6] Sydney Local Health District . Sydney Local Health District Strategic Plan 2018‐2023. 2018.

[7] Moore G , Du Toit A , Jameson B , et al. The Effectiveness of ‘Virtual Hospital’ Models of Care: A Rapid Evidence Scan. Sax Institute; 2020.

[8] Petit dit Dariel O , Cristofalo P . Improving patient safety in two French hospitals: why teamwork training is not enough. J Health Org Manag. 2020. In print. 10.1108/JHOM-02-2020-0045 32737962

[9] Australian Government Department of Health . Medicare Benefits Schedule Online, COVID‐19 Temporary. MBS Telehealth Services; 2020.

[10] NSW Clinical Excellence Commission . Application of PPE in Response to COVID‐19 Pandemic. 2020.

[11] Australian Government Department of Health . Advice to National Cabinet: The Australian Health Protection Principal Committee Recommended Special Provisions Be Applied to Vulnerable People in the Workplace. 2020.

[12] NSW Clinical Excellence Commission . Between the Flags. 2020. Accessed August 3, 2020.

[13] Australian Government Department of Health . Australian Health Protection Principal Committee (AHPPC) Coronavirus (COVID‐19). 2020.

[14] NSW Bureau of Health Information . Measurement Matters: Development of Patient Experience Key Performance Indicators for Local Health Districts in NSW. 2018. Accessed May 7, 2020.

